# Advances in Fabrication Technologies for the Development of Next-Generation Cardiovascular Stents

**DOI:** 10.3390/jfb14110544

**Published:** 2023-11-10

**Authors:** Ankita Das, Shreya Mehrotra, Ashok Kumar

**Affiliations:** 1 Department of Biological Sciences and Bioengineering, Indian Institute of Technology Kanpur, Kanpur 208016, UP, India; ankitad@iitk.ac.in; 2 Centre for Environmental Sciences and Engineering, Indian Institute of Technology Kanpur, Kanpur 208016, UP, India; 3 Centre of Excellence for Orthopaedics and Prosthetics, Gangwal School of Medical Sciences and Technology, Indian Institute of Technology Kanpur, Kanpur 208016, UP, India; 4 The Mehta Family Centre for Engineering in Medicine, Indian Institute of Technology Kanpur, Kanpur 208016, UP, India

**Keywords:** atherosclerosis, coronary artery disease, stent, bioresorbable, 3D printing

## Abstract

Coronary artery disease is the most prevalent cardiovascular disease, claiming millions of lives annually around the world. The current treatment includes surgically inserting a tubular construct, called a stent, inside arteries to restore blood flow. However, due to lack of patient-specific design, the commercial products cannot be used with different vessel anatomies. In this review, we have summarized the drawbacks in existing commercial metal stents which face problems of restenosis and inflammatory responses, owing to the development of neointimal hyperplasia. Further, we have highlighted the fabrication of stents using biodegradable polymers, which can circumvent most of the existing limitations. In this regard, we elaborated on the utilization of new fabrication methodologies based on additive manufacturing such as three-dimensional printing to design patient-specific stents. Finally, we have discussed the functionalization of these stent surfaces with suitable bioactive molecules which can prove to enhance their properties in preventing thrombosis and better healing of injured blood vessel lining.

## 1. Introduction

Cardiovascular diseases are the primary cause of death globally, accounting for approximately 32% of all deaths [1,2]. Among these, coronary artery disease (CAD) is one of the most prevalent cardiovascular diseases, resulting in substantially more deaths than cancer, respiratory diseases, and diabetes, thus obtruding a major health and economic burden on most developed nations. The deposition of fatty substances, cholesterol, cellular waste materials, calcium, and fibrin on the walls of blood arteries causes CAD. It subsequently advances towards plaque formation and blockage of blood vessels, which is termed atherosclerosis. The blockage of coronary arteries due to the deposition of fatty tissue developed on the walls of arteries results in reduced blood flow, thus causing damage to heart muscles. This also results in hypertension, further accompanied by angina in patients. Several risk factors such as age, obesity, smoking, sedentary lifestyle, and diet, as well as existing disease conditions like diabetes, non-alcoholic fatty liver disease, and hypertension contribute towards this pathological condition. Evidence also suggests that changes in blood parameters including increased levels of triglycerides (normal range < 150 mg/dL) and low-density lipoprotein (LDL; (normal range < 100 mg/dL)) with decreased levels of high-density lipoprotein (HDL; normal range > 40 mg/mL) [3] can also make a person more prone to developing CAD [4].

**Figure 1:**
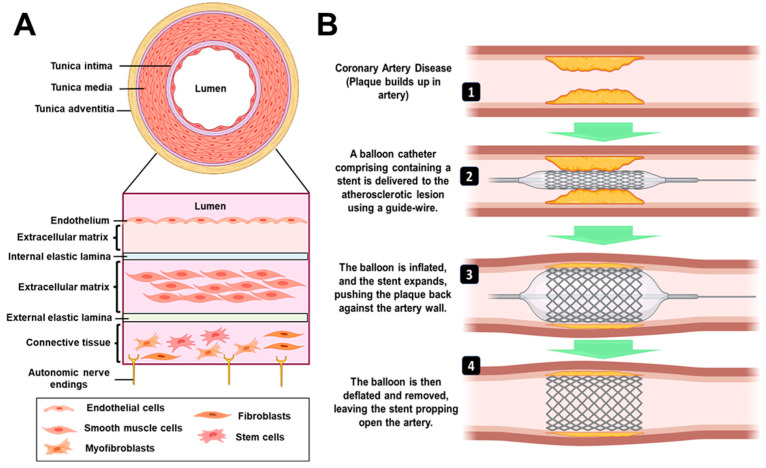
(**A**) Architecture of a blood vessel. Endothelial cells positioned in a single layer on the subendothelial extracellular matrix and internal elastic lamina make up the tunica intima. The external elastic lamina, extracellular matrix, and smooth muscle cells make up the tunica media. Fibroblasts and stem/progenitor cells are distributed throughout the connective tissue that makes up the tunica adventitia. (**B**) Procedure for percutaneous coronary intervention. It employs a catheter (a thin flexible tube) to place a stent to open up blood vessels. The balloon is initially inflated, followed by the expansion of the stent, which pushes the plaque against the artery wall. After the stent has been successfully implanted, the balloon is deflated and removed, leaving the stent to keep the artery open.

The typical anatomy of a blood vessel involves three layers: intima (innermost), media (middle layer), and adventitia (outermost) [5], as described in Figure 1A. With the onset of atherosclerosis, the intima is damaged due to plaque formation, leading to complications in blood flow through the vessels [6]. In a clinical setup, coronary artery blockages are predominantly treated by an invasive coronary artery bypass graft (CABG) surgery, in which an alternative artery or vein from the body is used to bypass the blocked part to resume proper circulation [7]. However, there are certain disadvantages associated with CABG that include harvesting of blood vessels from the body, the requirement of healthy patent conduits, degeneration of these grafts over time, and the requirement of longer recovery time [8]. The current gold standard treatment approach is percutaneous coronary intervention (PCI), which is a minimally invasive surgical procedure to treat stenosis (abnormal narrowing of blood vessels). The procedure relies on the use of a tiny catheter with a folded balloon on its tip which is threaded through the blocked artery. On reaching the blocked site, the balloon is inflated to compress the plaque against the wall of the artery (balloon angioplasty) [9]. According to the current ACS STEMI/NSTEMI guidelines published by the European Society of Cardiology in August 2023, there are not any specific randomized clinical trials that contrast surgical and percutaneous revascularization in acute coronary syndrome (ACS) patients [10]. Only in cases of ST-segment-elevated myocardial infarction (STEMI) when primary percutaneous coronary intervention (PPCI) is not practical—especially when there is persistent ischemia or a significant amount of compromised myocardium—should CABG be considered. Whenever possible, PCI is chosen over surgical treatment when patients need urgent revascularization in the context of very high-risk ST-segment-elevated ACS (NSTE-ACS), unless concurrent mechanical problems make surgical intervention the better option. The number of diseased vessels and the basic guidelines for cardiac revascularization should be taken into consideration when selecting a revascularization technique for other ACS patients. In the case of patients with multi-vessel diseases, the overall disease complexity and associated comorbidities will determine the choice of revascularization strategy.

In recent years, there have been modifications made to the surgical procedure that involves the incorporation of stents. These stents have proven to be effective in reducing the occurrence of abrupt vessel closure, preventing the re-accumulation of plaque and restenosis when compared to balloon angioplasty. Consequently, this has resulted in a decrease in the rate of target lesion revascularization (TLR) [11]. A stent is a mesh-type structure that is loaded over a guidewire, delivered via a balloon catheter to the site of the blocked artery, and deployed at the required site, as shown in Figure 1B. Different types of stents that are presently available in the market/clinics/research are categorized as: (1) bare metal stent, (2) drug-eluting stent, (3) bioengineered stent, (4) bioresorbable vascular scaffold (BVS), and (5) dual therapy stent. Some of the earliest stents were fabricated using metals (mostly stainless steel); however, they were associated with restenosis, i.e., re-narrowing of the blood vessels after a certain period [12]. This was mainly due to the migration and over-proliferation of smooth muscle cells from the intima layer to the blood vessel lining [6]. Further, they were also associated with an immunological response by the body and caused discomfort due to having a metal implant placed inside the artery for the patient’s lifetime. To combat the host immune response and reduce restenosis, new-generation stents incorporated/coated with drug molecules alone or drug molecules encapsulated within a polymeric material were developed (known as drug-eluting stents; DES) Such a method resulted in controlling the neointimal proliferation and reducing restenosis [13]. However, with time, the coating of the biodegradable polymer and drug was seen to wear off from the metal surface, leaving behind a bare metal stent (BMS) which had its own disadvantages as described above [14]. BVS is a non-metallic mesh tube that looks like a stent but progressively dissolves once the blocked artery can function normally. It became popular to overcome the issues with metallic stents. Such stents/scaffolds are made of resorbable polymers or metals that will degrade within a definite time point and hence will not remain in the body for a lifetime. On the other hand, bioengineered stents enabled the supply of such polymeric stents along with cells for a better healing process. Dual therapy stent is one of the advanced versions of stents in which, along with aspirin, an antiplatelet medicine like clopidogrel, prasugrel, or ticagrelor is given to the patient to control thrombosis [15]. While designing most of the advanced and new-generation bioresorbable stents, there are certain critical requirements which must be fulfilled. These are radial strength, thinner struts, biocompatibility, radio-opacity for X-ray and magnetic resonance imaging (MRI) visualization, acute and chronic recoil resistance, deliverability, lower crossing profile, and long-term integrity [16]. Further, some design considerations that need to be examined include the degradation and drug elution profile (incorporated within the stents), biocompatibility of degradable/non-degradable by-products, shelf-life, deterioration in the mechanical properties, and resorption time of the stent [17]. In lieu of the recent advancements in stent manufacturing and designing techniques, the use of advanced manufacturing/3D printing technology has emerged as a new direction that enables customized patient-specific implant fabrication.

The employment of modern technologies paves the way towards the development of next-generation stents; however, the coating of non-selective anti-proliferative drugs does not mitigate other adverse events like thrombosis, smooth muscle over-proliferation, and growth. In this regard, a functionalized stent surface can be utilized to avoid late thrombosis which is caused by the hampered endothelial cell growth in the intimal layer of the artery [18,19,20,21]. Thus, regeneration of the intimal endothelial cell layer is of utmost importance while designing potent implants to be used as cardiovascular stents. 

This review gives a comprehensive aspect on the state-of-the-art techniques that have translational potential for implant fabrication in treating cardiovascular diseases. It also considers the advancement in material design which is crucial for the fabrication of bioresorbable polymeric stents. We have described the studies involving different types of automated manufacturing technologies encompassing 3D printing, which are currently being experimented on by researchers to develop better quality stents. These techniques could reduce the time from bench to bedside and become a feasible option in the clinical setup.

## 2. Clinical Perspective of Stents

### 2.1. Metal Stents

The use of metals for cardiovascular devices dates to 1989 when the first coronary stent was implanted by Sigwart et al. [22]. Since then, several metal stents have become available in the global market, such as the Wallstent (manufactured by Boston Scientific Corporation) that utilizes stainless steel and SMART stents (manufactured by Cordis Corporation) which are composed of super-elastic metals like nickel and titanium [23]. These metal stents offer good longevity post-implantation but suffer from few adverse effects such as thrombosis and immunological response, thereby making them less suitable for long-term clinical implantation. To circumvent these problems, bio-corrodible metals like magnesium and iron have been explored for the fabrication of stents. Iron was the first metal to be used as a component in a bio-corrodible stent. Commercially available tubes were manufactured by Goodfellow, Cambridge, UK which are composed of around 99.8% iron and fabricated in a similar design as their stainless-steel counterparts [24]. However, improper corrosion rate observed in vivo hinders the application of these stents as biodegradable implants [25]. A second modified design brought about by the same company was the incorporation of other metals like aluminum, selenium, copper, manganese, and nickel [26]. Iron offers excellent mechanical properties (comparable to stainless steel counterparts), is radio-opaque, and therefore does not require additional markers to be detected by fluoroscopy. Magnesium is another biocompatible metal [27] which has been utilized for the fabrication of an AMS-1 bioresorbable stent, manufactured by Biotronik, Berlin, Germany. It is composed 93% of magnesium, manufactured by laser cutting and polished from the WE—43 magnesium alloy tube [28]. It has also been redesigned multiple times to alter the degradation rate and incorporate drug elution [29].

### 2.2. Drug-Eluting Stents

Drug-eluting stents involve the coating of bare metal stents by a polymeric matrix that elutes an active pharmacological agent such as anti-inflammatory drugs (sirolimus, everolimus, zotarolimus, and paclitaxel). The first DES implantation was performed with Cypher SES (sirolimus-eluting stent) that encountered a 0.0% rate of binary stenosis in comparison to patients who received BMS (26.6%) [30]. Almost during the same time, Taxus PES (paclitaxel-eluting stent) also gained regulatory approval. Satisfactory results were obtained (negligible rate of binary stenosis) with six months follow-up, which continued up to 4 years [31]. The details on the US Food and Drug Administration (FDA)-approved 1st and 2nd generation DES are summarized in Table 1.

### 2.3. Bioresorbable Vascular Scaffold

Next-generation alternatives involve the use of a bioresorbable vascular scaffold which is composed of biodegradable polymers. The first BVS approved by the FDA was Absorb GT1 in 2016. It was fabricated from poly(L-lactide) (PLLA) with a mixture of poly(D, L-lactide) (PDLLA) and 8.2 µg/mm of the anti-proliferative drug everolimus in equal amounts. A pair of radio-opaque platinum markers were also attached to the ends for visualization during coronary angiography [17]. Sirolimus and its synthetic variants are often employed as pharmaceutical agents in the context of DES and BVS. The description of the different drugs is given in Table 2. The polymers traditionally used for BVS include poly(lactic acid), poly(glycolic acid) [23], polycaprolactone [48], and chitosan [49,50]. A detailed description of the BVSs that have undergone and are still under clinical trials has been summarized in Table 3.

## 3. Limitations

The field of cardiovascular research has been progressing steadily with advancements in the fabrication of stents. The global stent market was estimated at USD 8.8 billion in 2021 and is expected to reach USD 12.3 billion by 2030. To match this increasing demand, significant research needs to be carried out, with emphasis on circumventing the currently existing limitations. In this regard, it must be mentioned that longer term follow-up of BMS showed high evidence of in-stent restenosis (ISR), around 20–30%, which was due to the migration of smooth muscle within the stents [78]. The rapid degradation (corrosion) is the biggest concern about magnesium and its alloys, which is responsible for the failure of its devices for cardiovascular applications [79,80]. After the Norwood procedure, a newborn was implanted with a Magmaris^®^ Resorbable Magnesium Scaffold (RMS) stent (BIOTRONIK AG, Bülach, Switzerland) to alleviate severe stenosis of the left pulmonary artery. However, the stent collapsed prematurely, according to a recent case study [81]. On the other hand, in spite of the favorable properties of metals for use in stent design, they come with problems of diminished late vessel healing and recovery of function. Thrombosis and a more extended period of dual antiplatelet therapy (DAPT) also become unfavorable factors in the case of DES. In the clinic, drug-eluting coatings of Cypher and Taxus are primarily designed to inhibit vascular smooth muscle cell (VSMC) proliferation. Nonetheless, the growth of endothelial cells (EC) is also inhibited by these medications, resulting in delayed re-endothelialization [82]. The first FDA-approved Absorb GT1 was discontinued as of 2017, owing to low sales. The stent has the potential to be employed in vessels with a diameter ranging from 2.5 mm to 3.75 mm; however, its application is not feasible outside of this range. Hence, the increased incidence in adverse events after a two-year study for Absorb III was due to the enrollment of patients with very small vessels (<2.25 mm) [83]. Despite the availability of commercial stents with varying dimensions (ranging from 8 to 60 mm in length and 2 to 4 mm in diameter), their standardized production does not account for specific patient characteristics. Consequently, the efficacy of these stents in treating different patients may not be uniform [84]. Since these concerns pose a challenge, personalized stents should be fabricated, which offer a lower risk of restenosis. Hence, designing a stent specific to the vessel anatomies of the patient can be a driving motive for future studies. The term “patient-specific stents” stands in the context of variable strut thickness, where thinner struts can be used in unstressed areas to avoid endoluminal paving, and stronger struts in stressed areas to avoid strut breaking. Similarly, flexibility in the overall stent design and each individual strut may provide for the best possible blood flow profile. For this purpose, multidetector cardiac computed tomography can offer images of vessel architecture in a non-invasive manner and accordingly, the stents can be designed as per the varying vessel geometries. The authors agree that these customized designs need to be approved by the FDA and similar regulatory bodies for clinical use. Recently, the FDA has approved patient-specific 3D-printed airway stents developed at the Cleveland Clinic. In a study published by the group, the researchers assessed the efficacy of nine 3D-printed airway stents over 4 years and compared the CT images taken before and after implantation of the stents [85]. Hence, similar studies with 3D-printed coronary stents can be proposed. With all this information considered, there is still an unrequited need to develop patient-specific stents, which have stable mechanical properties, minimal occurrence of thrombosis, and are effective in expediting endothelial healing.

## 4. Fabrication Technologies

The Absorb BVS is manufactured by melting PLLA resin and then extruding it into cylinders with thick walls and a small diameter. By heating the tubes, they expand into tubes with thinner walls and a larger diameter [86]. Thus, the traditional fabrication method for BVS is laser machining of polymer tubes; however, polymer properties are altered due to chemical and thermal effects or the molding processes, which poses to be a limitation [87]. To address the unique and challenging stent geometries specific to each patient, additive manufacturing technologies such as 3D printing (extrusion, solvent-cast, and light-based) can be employed.

### 4.1. 3D Printing

**Figure 2:**
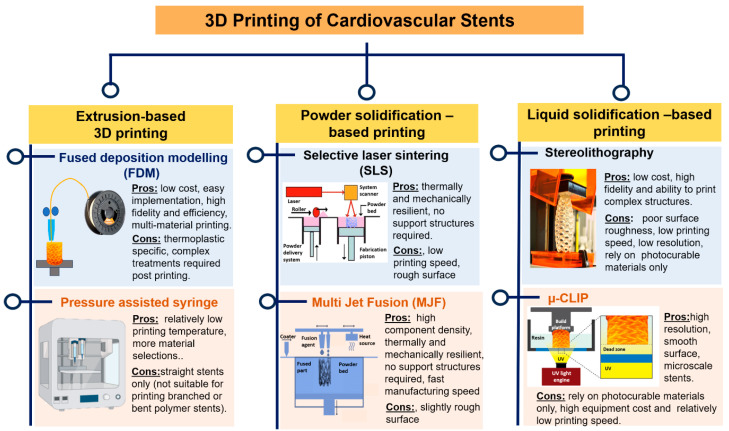
3D printing techniques utilized for the fabrication of cardiovascular stents.

The process of 3D printing involves the creation of three-dimensional things through the sequential deposition of polymeric material, based on a predetermined design generated using computer-aided design software. This technology is primarily utilized for quick prototyping purposes. It is an automated process which requires less time and is thus more efficient than conventional injection molding [88]. Therefore, it is widely used for the manufacturing of industrial and medical products [89]. This method offers many advantages for medical applications, including customized production, high precision, and fast fabrication [90]. Several techniques such as fused deposition modelling (FDM), selective laser sintering (SLS), and stereolithography (SLA) have been adopted for 3D printing [91,92]. The different 3D printing techniques along with their advantages and limitations have been elaborated in Figure 2.

#### 4.1.1. Extrusion-Based 3D Printing

##### Fused Deposition Modelling

Among all the 3D printing techniques, fused deposition modelling (FDM) is the most cost-effective and readily used [93,94]. Materials are initially manufactured into filaments and fed into a heating nozzle for filament-based printing. Later, this filament is melted, extruded, and placed onto a platform to create a three-dimensional structure layer-by-layer [95]. Therefore, the utilization of raw materials is utmost, and this fabrication process does not require any pre-requisite mold or template [96,97].

**Figure 3:**
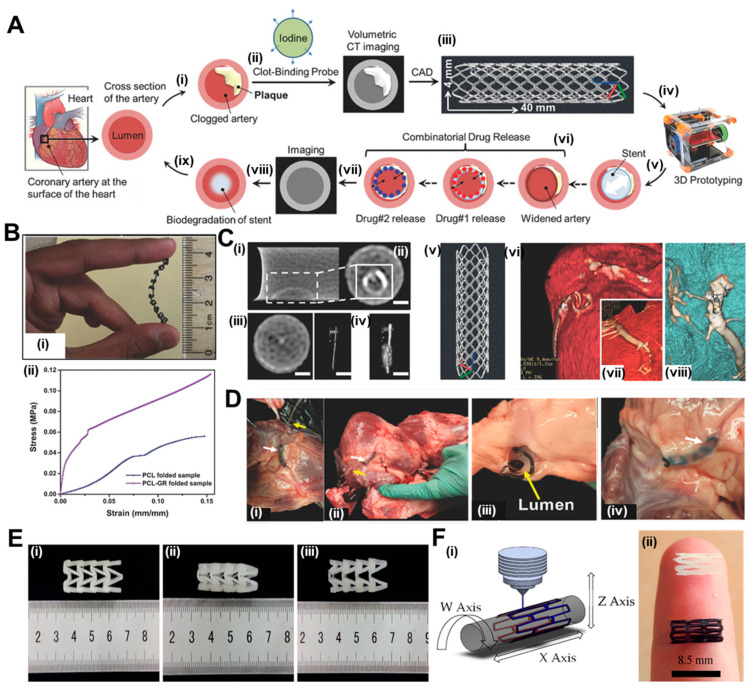
(**A**) (**i**) The presented image depicts a cross-section of an artery situated on the surface of the heart and plaque accumulation inside the lumen; (**ii**) A fibrin-targeted iodinated CT contrast probe is used to locate the blood clot and meanwhile, volumetric CT imaging accurately measures the obstruction; (**iii**) Imaging data is used to design a personalized stent using the CAD software; (**iv**) PCL–GR polymer composite is printed using an FDM; (**v**) Prototyped stent is inserted inside the artery; (**vi**) Two drugs are added for sequential release; (**vii**) CT imaging monitors healing process and (**vii**,**ix**) Biodegradation of polymer occurs within a wider artery. (**B**) (**i**) Flexible stent (4 cm × 0.4 cm in dimensions) printed using a customized extrusion setup and (**ii**) Mechanical properties of 3D-printed PCL and PCL–GR stents expressed as a stress–strain curve. (**C**) (**i**,**ii**) Cross-sectional CT images showing clot treated with targeted probe, scale bar—0.75 μm; (**iii**,**iv**) Cross-sectional CT images showing clot treated with non-targeted probe, scale bar—0.75 μm; (**v**) 3D printed PCL-GR stent and (**vi**–**viii**) CT scanning of pig heart deployed with PCL-GR stent. (**D**) (**i**–**iv**) Description of the steps involved in implanting a 3D-printed PCL–GR stent in an ex vivo pig artery model. White arrow marks 3D printed PCL-GR stent and yellow arrow denotes catheter for stent delivery. Reproduced with permission from [98] and (**E**) Shape recovery of PLA stents, (**i**) original stent, (**ii**) crimped stent, (**iii**) recovered stent. Reproduced from [100] and (**F**) (**i**) machine methodology and (**ii**) 3D-printed PCL (white) and PLA stents (black). Reproduced from [106].

Misra et al. [98] have utilized this technique to print a flat stent with the traditional polymer PCL and incorporated graphene nanoplatelets to generate a nanocomposite. The overview of their work has been summarized in Figure 3A. A fibrin-targeting probe was firstly devised to locate the clot in clogged arteries through computed tomography (CT) and accordingly, a computer-aided design (CAD) model for the stent to be printed (40 mm in length and 4 mm wide) was prepared. The printed stent exhibited adequate flexibility as shown in Figure 3B(i) and later folded over a mandrel (diameter 2 mm) to achieve the cylindrical structure. Dynamic mechanical analysis represented in Figure 3B(ii) shows a 22% improvement in mechanical strength, with Young’s modulus increasing from 726 ± 50 kPa in PCL stents in comparison to 889 ± 76 kPa for graphene-incorporated PCL stents. These results corroborated with the existing literature where graphene has been reported to increase the mechanical properties of materials [48]. The efficiency of the clot-targeting probe was revealed in cross-sectional CT images where the clot treated with the targeted probe showed high contrast in Figure 3C(i,ii), in comparison with the control solution which produced negligible contrast in Figure 3C(iii,iv). The structure of the graphene-containing PCL stent is shown in Figure 3C(v). Finally, the authors performed an ex vivo feasibility experiment in a swine model because of the high resemblance of pig hearts with human hearts and similar physical dimensions of the coronary arteries [99]. After sacrificing a six-month-old swine, the interior dimensions of the coronary artery were determined using a CT scan of the pig’s heart, prior to deploying the stent. The 3D-printed stent was implanted at the desired location, followed by CT scanning to corroborate the expansion of the artery as shown in Figure 3C(vi–viii). In the stenting process, the artery was first inflated with a stent loaded on a catheter, and then the stent was moved into the artery with the help of the catheter, as shown step-wise in Figure 3D(i–iv).

Polyesters such as poly(lactic acid) (PLA) and poly(glycolic acid) (PGA) are acceptable polymers for the fabrication of coronary stents. In a study by Jia et al. [100], PLA was used for the fabrication of self-expandable vascular stents to make use of the shape-memory properties of the polymer [101]. Shape-memory polymers can be used to fabricate stents because these could be self-deployed at body temperature without the need for any additional setup. Taking advantage of this property, 3D printing of the vascular stents was performed with a fused deposition modeling (FDM)-based printer with a nozzle diameter of 0.4 mm. The shape recovery of the stent was conducted at 70 °C in which it could revert to its initial shape within 5 s. The shape-memory property of PLA has also been explored by Wu et al. for the fabrication of stents by FDM with Negative Poisson’s Ratio (NPR), as shown in Figure 3E(i–iii) [102]. The effects of geometric parameters on wall thickness, radial compressive property of the stent, and stent diameter were studied. The changes in the dimensions of the diameter and length of the stent were calculated before and after the shape-memory experiment. When the deformation and recovery temperature were both kept at 65 ℃, then the diameter recovery reached to around 95% and the length was above 97%. The scientists discovered that increasing surface coverage and wall thickness and decreasing stent diameter increased implant radial force per unit length. Thus, this kind of material has a great future for self-expandable cardiovascular stent applications. However, stents generally undergo longitudinal foreshortening which means that the implant contracts in terms of its length when dilated. Hence, clinicians must use a stent longer in dimensions than the area to be stented in the artery where the plaque formation has occurred [103]. To circumvent this issue, Wang et al. [104] have developed a novel screw-extrusion-based 3D printing system that includes a mini-screw extruder and stents were fabricated with Zero Poisson’s Ratio (ZPR). The effects of surface morphology on the geometric and fabrication parameters were checked for the 3D-printed PCL stents. The cell viability of human umbilical vein cells on the stents was 90 ± 5%, which means that these implants can be utilized in vascular applications.

After promising results with PLA and PCL individually as polymers for BVS fabrication, another group used both materials to 3D print stents in a customized 3D printer. Hence, Guerra et al. had utilized a novel tubular 3D printer for the rapid manufacture of BVS which was based on FDM [105,106]. The machine methodology has been depicted in Figure 3F(i), while 3D-printed PCL and PLA stents are shown in Figure 3F(ii). Their previous results suggest that this technology could be utilized for BVS manufacturing as the group had managed to manufacture scaffolds under 5 min to achieve up to 85% precision [105]. A composite material comprising of PLA and PCL was prepared in a layered fashion with similar molecular weights of the polymers and the resulting blend improved the limitations with the individual material alone. PCL stents presented excellent expansion behavior but high recoil ratios; on the other hand, PLA stents presented excellent recoil ratio with inadequate radial expansion, due to their rigid nature. However, when composite stents were fabricated either with PLA or PCL as the inner layer, the implants possessed the properties of PCL stents (radial expansion) and PLA stents (recoil ratio). This approach could provide a good solution for bioresorbable scaffolds. The dynamical mechanical analysis shows that these polymers cannot be used alone with moduli either high or low, whereas the composite groups have values of modulus in the optimum range, making them ideal. Most importantly, the layer arrangement did not affect any of the mechanical property parameters. The composite PCL/PLA stents degraded almost moderately for all layer configurations, mostly owing to PLA degradation. The faster rate at which PLA breaks down would leave a frame made only of PCL in the end. PCL on the outside may be helpful because it may increase the growth of vascular cells. On the other hand, the internal PLA layer may slow the growth of cells, which may help control restenosis. This study was reported to the first work where the group had developed and presented composite PCL/PLA stents using a 3D printing process based on FDM that could comply with the strict BVS requirements [106].

**Figure 4:**
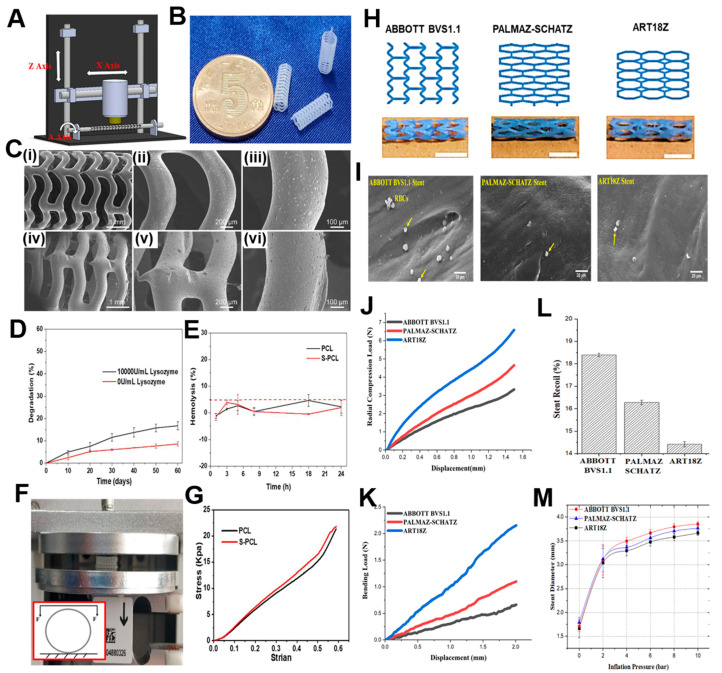
(**A**) Machine methodology of 3D printing. (**B**) 3D printed PCL stents. (**C**) Scanning electron micrographs of (**i**–**iii**) PCL stent and (**iv**–**vi**) S—PCL stent with different magnifications. (**D**) Degradation kinetics of) S—PCL stents in the presence and absence of lysozyme. (**E**) RBC hemolysis percentage after being exposed to PCL and S—PCL stent extracts at different time points. (**F**) Testing apparatus and inset showing a diagram for lateral crush resistance testing. (**G**) Stress–strain curve of PCL and S-PCL stents. Reproduced from [107]. (**H**) ABBOTT BVS1.1, PALMAZ-SCHATZ, and ART18Z, flat-shaped CAD models with finally printed stents, scale bar—5 mm. (**I**) Scanning electron micrographs depicting the adhesion of human blood platelets (yellow arrows) onto surface of stents printed using different designs. Diagram of the force–displacement relationship for (**J**) Radial compression test and (**K**) Three-point bending test. (**L**) Stent recoil expressed as a percentage and (**M**) Stent diameter while the balloon expands. Reproduced with permission from [114].

A similar version of a rotary 3D printing methodology (Figure 4A) was utilized by Qui et al. [107] for the fabrication of PCL stents (Figure 4B) which were later coated by 2-N, 6-O-sulfated chitosan (26SCS). Their 3D printing machine was based on electrospinning, where under a voltage of 4 kV, the PCL filaments were deposited onto a rotatory mandrel. The sulphated (S—PCL) and non-sulphated (PCL) stents were checked for morphology through scanning electron microscopy where the PCL stents exhibited a smooth surface in Figure 4C(i–iii) and the S—PCL stents showed a rough and porous surface in Figure 4C(iv–vi). This surface topography will ultimately lead to better endothelial cell proliferation [108] and play a major role in blood and cell compatibility. The degradation kinetics of the stents have been depicted in Figure 4D, where the weight loss of S—PCL was observed to be 16 and 7% in the presence and absence of lysozyme at 60 days, respectively. This result shows that PCL stents can be modified with appropriate non-toxic enzymes to accelerate their slow degradation rate, which may otherwise pose a limitation for using the polymer. A material is considered to be non-hemolytic if the rate of hemolysis is less than 2% and acceptable if the rate is between 2–5% [109]. Figure 4E shows that the hemolysis rate for all samples was below 5%, which suggested the hemocompatible property of both PCL and S—PCL stents. In this study, the in vivo loadings experienced by the stents were simulated by performing lateral crush resistance tests (Figure 4F). The mechanical properties were not altered after the 26SCS modification since no significant differences between the PCL and S—PCL stents were observed in the force–displacement and stress–strain curves (Figure 4G).

The degradation rate of a polymer is an important deciding factor for stent fabrication. Poly(p-dioxanone) (PPDO) is an aliphatic semi-crystalline polyester with ester and ether bonds and a resorption time of 6 months [110]. The material is biocompatible, bioresorbable, and flexible. The Food and Drug Administration has also authorized numerous PPDO medical implants, including sutures, clips, staples, pins, and mesh [111]. A recent study explored how the temperature of the nozzle, the temperature of the bed, the thickness of the layers, and the speed of printing affect the mechanical features of PPDO stents [112]. The enhancement of Young’s modulus and yield strength of PPDO FDM parts were observed with an increase in nozzle temperature and bed temperature. The same polymer has been used by another group to fabricate stents using FDM by using poly(dioxanone) (PDO) and PLA blends, and the authors implemented a virtual testing system to examine the structural response of custom 3D-printed bioresorbable stent designs [113]. Virtual testing is crucial for studying stent mechanics in an in-silico scenario, avoiding costly animal studies and trial-and-error methods.

##### Solvent Cast 3D Printing

For melt-based techniques, processing pressures and temperatures need to be maintained at a high value so that continuous flow is maintained. This presents a limitation because the type of polymer that can be utilized for printing becomes restricted. A second type of extrusion-based printing is solvent-cast 3D printing (SC-3DP), which offers an alternative approach to print a wider range of polymers. In this case, polymers are first dissolved in a volatile solvent and while a solid polymer filament gets deposited, the solvent gets evaporated. However, since stents have many interconnected mesh structures, printing on a flat substrate by applying this technique becomes challenging. Therefore, a cylindrical substrate could overcome these difficulties while developing SC-3DP. This technique was utilized by Singh et al. [114] where they had utilized polycaprolactone stents reinforced with carbonyl iron powder (CIP) according to previously existing designs such as ABBOTT BVS 1.1, PALMA-SCHATZ, and ART18Z. The CAD designs and the printed stents have been shown in Figure 4H. The scanning electron micrographs show that the stents possess excellent hemocompatibility as seen by the inactivated adhesion of platelets in all the three types, although the least adherence was noticed in the PALMAZ-SCHATZ stent (Figure 4I). The ART18Z stents and ABBOTT BVS 1.1 displayed a higher (6.59 ± 0.52 N) and lower (3.32 ± 0.36 N) value of radial compression force, respectively, with a moderate value (4.65 ± 0.21 N) for the PALMAZ-SCHATZ stent (Figure 4J). The flexibility of the PALMAZ-SCHATZ stent was also moderate compared to the ART18Z stents and ABBOTT BVS1.1 (Figure 4K). This radial compressive force is inversely related to the stent’s recoil ratio, since stents with the highest radial compression force will experience the least recoil, as shown in Figure 4L. These results are also corroborating with the stent diameter calculated after over-expansion, since ART18Z expanded the least on over-inflating with a balloon (Figure 4M). The enhancement of this hemocompatibility and mechanical properties could be attributed to the addition of 1 wt% of CIP in the PCL matrix [115]. Overall, the results concluded that the stents printed with the PALMAZ-SCHATZ design had better biological and mechanical properties than ABBOTT BVS1.1 and ART18Z that could be used in the treatment of stenotic arteries. An advancement to this work was carried out by the same group where the effects of process parameters on the flexibility of stents, radial compression load, and percentage reduction in strut width and thickness, were evaluated [116]. The printing speed and layer thickness were observed to be important factors and contributed to the flexibility, radial compression load, and reduction in strut width and thickness of the composite stents. The interaction of layer thickness and printing speed also influenced the mechanical properties of the printed stents. It was observed that at a slow printing speed and small layer thickness, the maximum load for bending and radial compression load were obtained. The optimized process parameters were 1.08% CIP concentration, layer thickness around 0.2 mm, and printing speed of 8.02 mm/s. By following the parameters, the output responses were observed to be 1.13 N load for bending, 4.12 N radial compression load, 12.80% reduction in strut width, and 1.90% reduction in strut thickness. For future work, these optimized parameters would be beneficial in the design and fabrication of stents with varied topologies.

#### 4.1.2. Light-Based Printing

**Figure 5:**
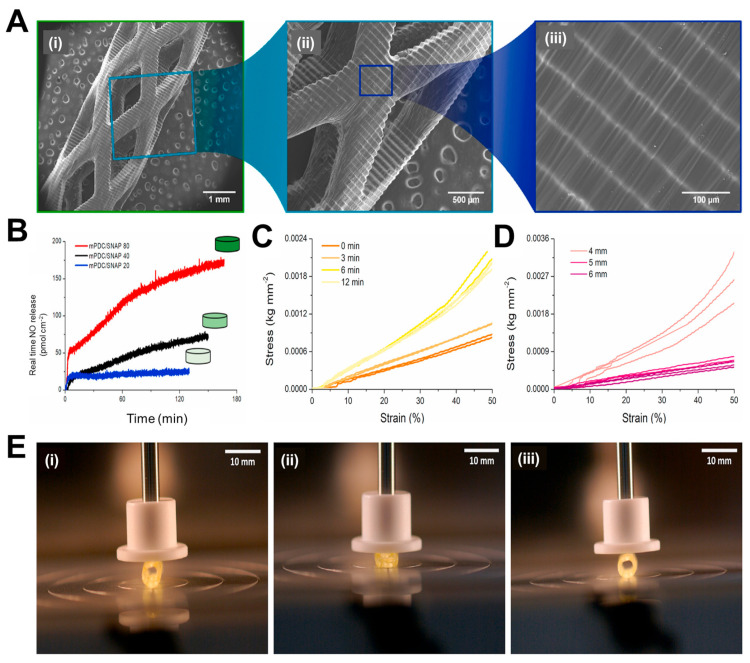
(**A**) (**i**–**iii**) Scanning electron micrographs of 3D printed stents with increases in magnification from left to right, demonstrating the printing accuracy from the millimeter to the micrometer length scale. (**B**) Real—time NO released by 3D-printed mPDC/80, 40, and 20 discs, after being immersed in PBS (pH 7.4) at 37 °C. Stress—strain compression curves for (**C**) uncured and post—cured mPDC stents at 3, 6, and 12 min, and (**D**) mPDC stents with 4, 5, and 6 mm diameters post—cured for 3 min and (**E**) Images of a 6 mm stent (**i**) before, (**ii**) fully compressed, and (**iii**) extended after compression. Reproduced with permission from [122].

While various research groups were working on the traditional polymers, Lith et al. [117] had thought of developing a technology for on-the-spot fabrication of high-resolution bioresorbable vascular stents based on a versatile form of additive manufacturing technology that will be entirely based on patient-specific design. In their endeavors, a four-step process will be followed for the placement of stents in patients: (1) vascular parameters of the patient to be assessed with imaging techniques; (2) the design of the stent to be made with computer-aided software based on patient-specific parameters; (3) the stent is fabricated on-the-spot by 3D printing following the design; and (4) the customized stent can now be utilized for implantation in the patient. The authors had utilized an antioxidant, photocurable, and bioresorbable citrate-based biomaterial and printing was carried out using a custom-made micro-continuous liquid interface production system (mCLIP), a technology already described by Tumbleston et al. [118]. It is based on the principle that oxygen can either quench the photoexcited photo initiator or create peroxides by combining with the free radical from the photo initiator, creating a “dead zone” that cannot be photocured. If stereolithography is conducted above an oxygen-permeable window, a continuous liquid interface will be produced by a thin liquid layer, which will be due to oxygen contact between the window and the cured part. Previously, Park’s group had used bio-plotting that does not accommodate stent customization [119], whereas Flege’s BVS exhibited resolution and poor surface finish [120]. Most importantly, both techniques require fabrication times of 10 h or more. On the other hand, this new mCLIP technology was a continuous process that could speed up the printing process tremendously as compared to FDM that works through layer-by-layer assembly. Hence, this group introduced the first successful mCLIP-produced, antioxidant, and customizable BVS that is similar to the mechanical strength of the nitinol stent. The group used methacrylated polydiolcitrates which were photo-cured, and intrinsic antioxidant properties were also utilized to chelate trace transition metals that could be potentially harmful. This property was lacking in the conventional polymers used in 3D printing. This work was further optimized by Ware et al. [121] in which they could fabricate a 2 cm tall vascular stent comprising of 4000 layers in 26.5 min. In another study by Oliveira et al. [122], the same polymer has been utilized for printing BVS with another light-assisted 3D printing technique, known as digital light processing (DLP). Commercial low-cost DLP printers also provide good resolution, which are comparable to expensive 3D printers that employ mCLIP and projection micro-stereolithography techniques. Nitric oxide (NO) is released by endothelial cells which inhibits the adhesion of platelets, maintains vascular tone, and inhibits SMC proliferation [123]. The researchers employed the nitric oxide (NO) donor known as S-nitroso-N-acetyl-d-penicillamine (SNAP) and integrated it into the methacrylated poly(dodecanediol citrate) (mPDC) scaffolds via absorption. The SEM images show the ultrastructure of the stent (3 mm in diameter) with increasing magnification (Figure 5A(i–iii)), where Figure 5A(i) shows that the stent has a consistent geometric structure characterized by clearly defined boundaries and a textured surface with indentations. The layers exhibit strong adhesion and maintain their structural integrity without any surface imperfections, as depicted in Figure 5A(ii,iii). Since polyesters hydrolyze slowly under physiological conditions, taking over 3 years, the mPDC stents were subjected to accelerated degradation (temperature 60 °C and pH 12) and percentage mass loss and change in pH have been shown for 21 days. The mass loss reached 20% after 5 days of rapid degradation, remained stable until day 10, then increased to 60% by day 15, and remained consistent throughout the experiment. This mass loss pattern indicates a two-step hydrolytic breakdown process. During mPDC hydrolysis, the pH fluctuation reduces practically linearly towards zero due to the partial neutralization of the basic solution (pH 12) by free citric acid. Further, after approximately 2 h, different variants of mPDC/SNAP scaffolds attain a constant NO release rate and this value falls within the range of NO release from healthy endothelial cells (Figure 5B). The mechanical properties of the stents were further evaluated, where Figure 5C presents the compressive stress–strain curves derived from the compression testing of 3D-printed stents with a diameter of 6 mm. These stents were tested in their uncured state and subsequently subjected to post-cure periods of 3, 6, and 12 min. The viscoelastic behavior of stents is evident when subjected to linear compressions up to 50% of their original diameter, a property commonly observed in elastomeric materials. The optimal post-cure irradiation time for the current mPDC stents was determined to be 3 min. This duration resulted in consistent stress–strain behavior and an elastic response of up to 50% strain. This elasticity enables the stent structure to recover after deployment. On the other hand, Figure 5D illustrates the stress–strain behavior of stents with diameters of 4 mm, 5 mm, and 6 mm that underwent a post-cure period of 3 min. In a similar manner, it was observed that stents with diameters ranging from 5 to 6 mm, subjected to a post-cure duration of 3 min, exhibited superior compressive capabilities compared to stents with a diameter of 4 mm, for the intended use. It can also be seen that the stent could transform into a fully collapsed state on compression and after removal of the mechanical load, it immediately reverted back to its original expanded state, as represented in Figure 5E(i–iii). Thus, these stents are an advancement in material design for the fabrication of bioresorbable vascular stents, where NO release will play an important role as a therapeutic entity. However, there were some shortcomings of these studies, such as the materials used for fabricating the stents are not FDA-approved and hence require extensive studies before they can be taken further.

#### 4.1.3. Selective Laser Melting

Selective laser melting is another important 3D printing technique, wherein, according to predetermined trajectories, a laser or electron beam spot travels across a powder bed’s surface. When the spot moves away from the exposed powders, the powders absorb enough thermal energy to change into a molten state, which fuses or sinters the material together. After that, a levelling roller is used to add another layer of powder on top of the first powder bed. Subsequently, the powders in the current layer are selectively sintered with those in the preceding layer(s) using the laser or electron beam point. A 3D structure can thus be fabricated in a layer-by-layer fashion using this technique. Currently, this technique has been used for coronary stent prototype fabrication using poly(L-lactic acid) (PLLA) and poly(L-lactic acid co- poly-ε-caprolactone (70:30) (PLLA-co-PCL) [120]. Three criteria related to the 3D printing process that are known to impact the thickness of sintered objects were evaluated: the number of repeated scans, the laser beam diameter, and the laser intensity. The prototypes were prepared with an inner diameter of 3 mm and a height of 15 mm. The biocompatibility assay showed that the stents enhanced the proliferation of endothelial cells and decreased the viability of smooth muscle cells, thereby controlling the issue of neointimal hyperplasia which leads to stenosis. When compared to PLLA-co-PCL, PLLA was determined to be better for the application since it produced high detail resolution and had a more dependable, longer lasting shelf-life. Nonetheless, further information is needed about the in vivo biodegradation and biocompatibility of prospective stents exposed to gamma irradiation, with a focus on the induction of inflammation.

Despite such promising results, it should be mentioned that the non-uniform degradability of the biodegradable stents may lead to vessel occlusion and thrombosis. It has also been reported that the degradation rate of the stents can lead to improper vessel remodeling. For example, a study has shown that the fast resorption rate of the stents has led to inflammation and granuloma formation in spite of good angiographic results [23]. Hence, the biodegradable stent has to be designed in a way that the material undergoes bulk erosion slowly (coating with slowly degrading polymers) and not degrade non-uniformly, thus creating pieces of varying sizes.

## 5. Functionalization with Bioactive Molecules

**Figure 6:**
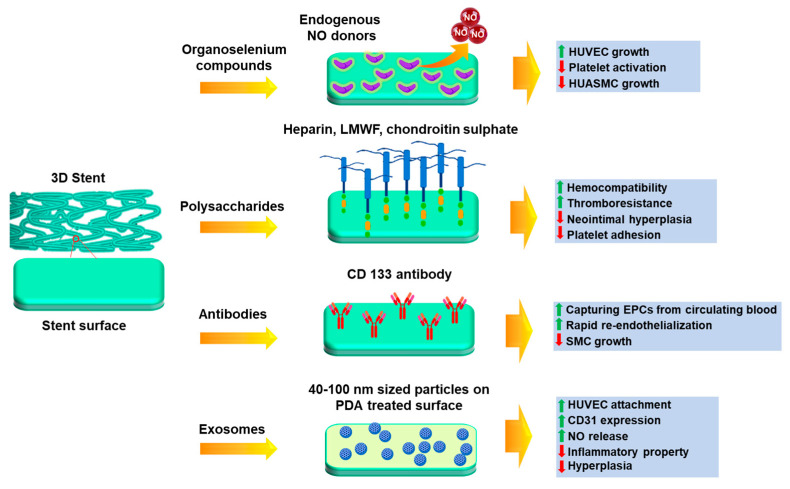
Different approaches for the surface modification of stents to enhance endothelialization.

Endothelium health is regarded as the most important component of a robust vasculature. It preserves the vascular cells’ fibrinolytic, antiplatelet, and anticoagulant characteristics. The healthy endothelium produces a wide range of factors that regulate vascular tone, cellular adhesion, thromboresistance, smooth muscle cell proliferation, and vessel wall inflammation [124,125]. Hence delayed re-endothelialization of vessels becomes a major drawback of DES, due to the usage of non-specific cytotoxic drugs. Thus, various research groups have been trying to modulate the surface of stents to optimize the growth of endothelial cells, as seen in Figure 6. Among them, the incorporation of bioactive molecules, antibodies, etc., can prove to be a solution. For rapid endothelial lining repair, nitric-oxide-releasing compounds can be a good option, since NO is identified as an endothelium-derived relaxing factor. It is produced by the conversion of L-arginine by endothelial nitric oxide synthase (eNOS). NO activates guanylate cyclase in smooth muscle cells and takes part in cGMP-mediated vasodilation [126,127]. Further, it is reported that organoselenium compounds like 3,3-diselenodipropionic acid (SeDPA) and selenocystamine (SeCA) can generate NO from endogenous donors [128]. Hence Yang et al. [129] had immobilized SePDA on the SS 316L surface via plasma polymerized allylamine coating. The coating with NO showed significant inhibitory effects on the proliferation and migration of smooth muscle cells (SMCs) and collagen-induced platelet activation with enhanced endothelial cell migration and growth. In addition, the material displayed a high degree of cell selectivity by favoring the proliferation of HUVECs over SMCs. The in vivo results also demonstrated that this coating provided an NO-rich microenvironment that would be beneficial in the healing of the damaged endothelial lining in contact with the lumen of the stent.

Several polysaccharides, glycosaminoglycans, and extracellular matrix proteins have been immobilized for better endothelial cell growth, such as sulphated polysaccharides like heparin, fucoidan [130,131,132,133], chondroitin sulphate [134,135], and non−sulphated polysaccharide like hyaluronic acid [136]. Heparin is a sulfated glycosaminoglycan that is often used as an anticoagulant drug coating on metal stents to prevent thrombosis [137] and is an anti−inflammatory medication used to treat obstructive bronchopulmonary disease [138]. Lee et al. [139] immobilized heparin on 3D printed PLA stents using EDC-NHS chemistry. The results depict that the developed stent showed excellent anticoagulant activity, such as thromboresistance and hemocompatibility. The modulation of endothelial cell and smooth muscle cell proliferation was also maintained. The in vivo results show that the heparin−coated stents supported the widest lumen area and minimal neointimal hyperplasia. There were also no incidences of coagulation or thrombosis. In another study by Liu et al. [140], a novel bifurcated stent graft (BSG) was devised using textile forming technology in which they had coated the surface with silk fibroin (SF)—encapsulated heparin by dip coating. In vitro results showed that for the steam treated BSG, the sustained release of heparin was achieved up to 120 h in comparison to the air-dried samples. This could be due to the structural transition of SF during steam treatment which affects the release pattern of heparin. The viability of SMCs cultured was significantly inhibited at 144 h in the presence of the release medium of the steam treated BSG. This duration was longer than that for the air−dried BSG, which confirmed the sustained release of heparin from the implant.

Endothelial progenitor cells (EPCs) have been identified as a contributing factor to re-endothelialization. They are an anchoring type of cell line which are found to be circulating in peripheral blood [141]. Keeping this mind, Wawrzyńska et al. [142] have immobilized the CD133 antibody on the SS 316L surface and revealed that EPCs adhere better to anti−CD133−coated surfaces than anti-VEGFR and anti−CD34 antibodies [143,144]. A short animal toxicity investigation found that the stent surface coating for surface functionalization was safe at macro and nano levels, but more in vitro and in vivo studies are needed. Endothelial cell adhesive peptides can be also immobilized on stent surfaces to enhance endothelial cell response and hemocompatibility [145]. The synthesis of linear sequence peptides RGDS and YIGSR have been accomplished, along with the development of a dual platform that incorporates both motifs within a single biomolecule. Compared to control samples, cell adhesion assays revealed a marked increase in the number of adherent cells and their distribution across functionalized films.

It has been previously reported that the exosome-involved exchange of genetic information results in neovascularization [146]. Hence, a study was conducted by Hou et al. [147] where positively charged exosomes were immobilized on negatively charged poly-dopamine-treated SS 316L surfaces by electrostatic assembly. An exosome is a natural nanoparticle (40−100 nm in diameter) that contains complex proteins and RNAs, and under blood flow acting, it causes artificial cells to express CD31, which is specific factor for vascular endothelial cells [148,149]. Cell culture experiments by these groups proved that the immobilized exosomes were able to improve HUVEC migration and proliferation, NO release, and CD31 expression, which confirmed its role in aiding endothelialization. It was also found that the attached macrophages were transformed from the M1 phenotype to the M2 phenotype in the presence of the exosome-modified surface, which suggested its anti-inflammation property. Scaffolds implanted in the carotid artery of rats also showed good anti-hyperplasia functions due to the coating. Thus, this approach has the ability to be further explored to promote the regeneration of endothelial tissue.

## 6. Conclusions

Major disadvantages of restenosis, thrombosis, inflammation, and delayed re-endothelialization have been seen in both metal and drug−eluting stents. Such issues can be tackled by utilizing bioresorbable stents and functionalizing them with bioactive molecules. The current research suggests that advanced technologies like 3D printing have proven to be successful in the fabrication of patient−specific stents and have also achieved mechanically strong implants designed from low moduli polymers. Customized stents were prepared firstly by assessing vessel anatomy through imaging techniques and then 3D printing them as per the patient’s requirements. Self−expandable stents made from shape−memory polymers have also proved to be useful since they can recover to their initial shape by heating. Multi-layered stents can be used where the different layers can perform separate functions like maintaining rigidity, resisting recoil, etc. 3D printing mainly deals with the development of high−resolution customized vascular implants in a shorter duration of time. The degradation rate of the stents can be modulated by varying the composition of polymers, which in turn also affects vascular compatibility. Finally, the problem of endothelial lining repair can be circumvented by immobilizing anticoagulants, polysaccharides, EPC−capturing antibodies, and exosomes. Thus, it can be concluded that these next-generation fabrication methods have great potential to make improved coronary stents with ideal properties.

## 7. Future Perspectives

There are still certain aspects which need better evaluation that include in vitro cytocompatibility and hemocompatibility in cases where the implants have been characterized only for their physical and mechanical behavior. On the other hand, scaffolds which have been well characterized in vitro should advance towards preclinical experiments in small and large animal models. There is also a lot of scope for material design which could prove to be beneficial in terms of mechanical stability and biocompatibility. Such dual favorable properties will prove to be quite beneficial for the increasing demand in this field. Hence, a brief overview of the existing advanced technologies described in this review will be beneficial in designing patient-specific next-generation stents.

## Figures and Tables

**Table 1:** List of FDA-approved drug-eluting stents.

Stent	Manufacturer	Base Material	Strut Thickness (µm)	Drug Name and Conc. (µg/cm^2^)	Polymer for Drug Coating	Polymer Thickness (µm)	Drug Release	Ref.
Cypher (1st Gen)	Cordis Corporation	SS	140	Sirolimus (140)	PEVA and PBMA	12.6	80%	[32,33]
Taxus Express (1st Gen)	Boston Scientific Corporation	SS	132	Paclitaxel (100)	SIBS	16.0	<10%	[34,35]
Taxus Liberté (1st Gen)	Boston Scientific Corporation	SS	97	Paclitaxel (100)	SIBS	16.0	<10%	[36,37]
Endeavour (2nd Gen)	Medtronic	CoCr	91	Zotarolimus (100)	PC	4.1	95%	[13,38,39,40,41]
Xience V (2nd Gen)	Abbott Laboratories	CoCr	81	Everolimus (100)	PVDF-HFP and PBMA	7.6	80%	[42,43,44,45,46,47]

Abbreviations: SS—stainless steel; CoCr—cobalt-chromium; PEVA—polyethylene-co-vinyl acetate; PBMA—poly(n-butyl methacrylate); SIBS—poly(styrene-b-isobutylene-b-styrene); PC—phosphorylcholine; PVDF—poly(vinylidene fluoride); HFP—hexafluoropropylene.

**Table 2:** Description of drugs used in drug-eluting stents and bioresorbable vascular scaffolds.

Drug	Description
Sirolimus (previously called rapamycin)	Macrolide antibiotic with immunosuppressant functions
Zotarolimus	Semisynthetic (made by substituting a tetrazole ring for the native hydroxyl group at position 42 in rapamycin)
Everolimus	Synthetic derivative of sirolimus (40-O-[2-hydroxyethyl]-rapamycin)
Paclitaxel	Antineoplastic agent

**Table 3:** Comparative details of bioresorbable vascular scaffolds.

Stent	Manufacturer	Base Material	Strut Thickness (µm)	Drug Name	Polymer for Drug Coating	Resorption Time (Months)	Ref.
ABSORB 1.0	Abbott Vascular	PLLA	150	Everolimus	PDLLA	24	[51]
ABSORB 1.1	Abbott Vascular	PLLA	150	Everolimus	PDLLA	24	[3,52]
DESolve150/ DESolve Nx	Elixir Medical	PLLA	150	Myolimus	PLLA	12	[53,54,55]
DESolve 100	Elixir Medical	PLLA	100	Novolimus	DESyne BD	-	[56,57]
DESolve Cx	Elixir Medical	-	120	Novolimus	-		[57]
REVA	Reva Medical Inc.	Tyrosine-derived polycarbonate	200	None	-	24	[55]
ReZolve	Reva Medical Inc.	ReZorb^TM^ polymer	115–230	Sirolimus	-	4–6	[55,58]
ReZolve 2	Reva Medical Inc.	ReZorb^TM^ polymer	-	Sirolimus	-	-	-
Fantom	Reva Medical Inc.	Desaminotyrosine polycarbonate	125	Sirolimus	ReZorbTM polymer	-	[59]
MeRes	Meril Life Sciences	PLA	>200	Sirolimus	Non-inflammatory biodegradable polymer	-	[60,61,62]
MeRes 100	Meril Life Sciences	PLLA	100	Sirolimus	PDLLA		[61]
FORTITUDE	Amaranth Medical, Inc.	High MW PLLA	150–200	Sirolimus	-	10	[55,63]
APTITUDE	Amaranth Medical, Inc.	Amorphous PLLA	115	Sirolimus	-	3–6	[64]
MAGNITUDE	Amaranth Medical, Inc.	PLLA	<100	-	-	24–36	-
XINSORB	Huaan Biotechnology Group Co., Ltd.	PLLA	160	Sirolimus	PDLLA/PLLA	-	[65,66,67,68]
IDEAL (1st Gen)	Bioabsorbable Therapeutics Inc.	Poly (anhydride ester) salicylic acid (SA)	200	Sirolimus	SA linked with adipic acid	9–12	[52]
IDEAL (2nd Gen)	Xenogenics Corporation	Poly (anhydride ester) salicylic acid (SA)	175	Sirolimus	-	>12	[51,69]
Mirage BRMS	Manli Cardiology	PLLA	125, 150	Sirolimus	PLA	14	[70,71]
Igaki-Tamai	Kyoto Medical Planning Co., Ltd.	PLLA	170	None	-	24	[3,72,73]
ArterioSorb	Arterius Ltd.	PLLA	95, 120	Sirolimus	PDLA		[73]
ART Pure	Arterial Remodelling Technologies Inc.	PLA	-	None	-	24	[74,75]
ON-AVS	OrbusNeich	PDLA	150	Sirolimus and CD34^+^	-	>6	[55,76]
Stanza BRS	480 Biomedical	PLGA	-	-	Polyester/ Poly-urethane elastomer	12	[23,77]

Abbreviations: PLA—poly(lactic acid), PLLA—poly(L- lactic acid), PDLA—poly(D- lactic acid), PDLLA—poly(D, L- lactic acid), PLGA—poly(lactic-co-glycolic acid).

## Data Availability

Not applicable.

## References

[1] Fox K.A.A., Metra M., Morais J., Atar D. (2020). The Myth of ‘Stable’ coronary artery disease. Nat. Rev. Cardiol..

[2] Benjamin E.J., Muntner P., Alonso A., Bittencourt M.S., Callaway C.W., Carson A.P., Chamberlain A.M., Chang A.R., Cheng S., Das S.R. (2019). Heart Disease and Stroke Statistics—2019 Update: A Report from the American Heart Association. Circulation.

[3] Iqbal J., Gunn J., Serruys P.W. (2013). Coronary Stents: Historical Development, Current Status and Future Directions. Br. Med. Bull..

[4] Ueda P., Gulayin P., Danaei G. (2018). Long-Term Moderately Elevated LDL-Cholesterol and Blood Pressure and Risk of Coronary Heart Disease. PLoS ONE.

[5] Kloc M., Ghobrial R.M. (2014). Chronic Allograft Rejection: A Significant Hurdle to Transplant Success. Burns Trauma.

[6] Madamanchi N.R., Vendrov A., Runge M.S. (2005). Oxidative Stress and Vascular Disease. Arterioscler. Thromb. Vasc. Biol..

[7] Braun M.M., Stevens W.A. (2018). Stable Coronary Artery Disease: Treatment. Am. Fam. Physician.

[8] Burgess S.N., John J., Juergens C.P., French J.K. (2015). Coronary Artery Bypass Grafting versus Percutaneous Intervention in Coronary Revascularization: A Historical Perspective and Review. Res. Rep. Clin. Cardiol..

[9] Neumann F.-J., Sousa-Uva M., Ahlsson A., Alfonso F., Banning A.P., Benedetto U., Byrne R.A., Collet J.-P., Falk V., Head S.J. (2019). 2018 ESC/EACTS Guidelines on Myocardial Revascularization. Eur. Heart J..

[10] Byrne R.A., Rossello X., Coughlan J.J., Barbato E., Berry C., Chieffo A., Claeys M.J., Dan G.-A., Dweck M.R., Galbraith M. (2023). 2023 ESC Guidelines for the Management of Acute Coronary Syndromes. Eur. Heart J..

[11] Lobato E.B., Kaplan J.A., Cronin B., Maus T.M. (2019). Chapter 3—Care of the Patient with Coronary Stents Undergoing Noncardiac Surgery. Essentials of Cardiac Anesthesia for Noncardiac Surgery.

[12] Li X., Zhang W., Lin W., Qiu H., Qi Y., Ma X., Qi H., He Y., Zhang H., Qian J. (2020). Long-Term Efficacy of Biodegradable Metal-Polymer Composite Stents after the First and the Second Implantations into Porcine Coronary Arteries. ACS Appl. Mater. Interfaces.

[13] Garg S., Serruys P.W. (2010). Coronary Stents: Current Status. J. Am. Coll. Cardiol..

[14] Joner M., Finn A.V., Farb A., Mont E.K., Kolodgie F.D., Ladich E., Kutys R., Skorija K., Gold H.K., Virmani R. (2006). Pathology of Drug-Eluting Stents in Humans: Delayed Healing and Late Thrombotic Risk. J. Am. Coll. Cardiol..

[15] Bates E.R., Lau W.C., Angiolillo D.J. (2011). Clopidogrel–Drug Interactions. J. Am. Coll. Cardiol..

[16] Menown I., Noad R., Garcia E., Meredith I. (2010). The Platinum Chromium Element Stent Platform: From Alloy, to Design, to Clinical Practice. Adv. Ther..

[17] McMahon S., Bertollo N., Cearbhaill E.D.O., Salber J., Pierucci L., Duffy P., Dürig T., Bi V., Wang W. (2018). Bio-Resorbable Polymer Stents: A Review of Material Progress and Prospects. Prog. Polym. Sci..

[18] Garg S., Serruys P.W. (2010). Coronary Stents: Looking Forward. J. Am. Coll. Cardiol..

[19] Windecker S., Meier B. (2007). Late Coronary Stent Thrombosis. Circulation.

[20] Finn A.V., John M.C., Gold H.K., Newell J., Nakazawa G., Joner M., Kolodgie F.D., Virmani R. (2007). Response to Letter Regarding Article, “Pathological Correlates of Late Drug-Eluting Stent Thrombosis: Strut Coverage as a Marker of Endothelialization”. Circulation.

[21] Pilgrim T., Windecker S. (2009). Drug-Eluting Stent Thrombosis. Minerva Cardioangiol..

[22] Sigwart U., Urban P., Golf S., Kaufmann U., Imbert C., Fischer A., Kappenberger L. (1988). Emergency Stenting for Acute Occlusion after Coronary Balloon Angioplasty. Circulation.

[23] Sharma U., Concagh D., Core L., Kuang Y., You C., Pham Q., Zugates G., Busold R., Webber S., Merlo J. (2018). The Development of Bioresorbable Composite Polymeric Implants with High Mechanical Strength. Nat. Mater..

[24] Peuster M., Wohlsein P., Brügmann M., Ehlerding M., Seidler K., Fink C., Brauer H., Fischer A., Hausdorf G. (2001). A Novel Approach to Temporary Stenting: Degradable Cardiovascular Stents Produced from Corrodible Metal-Results 6–18 Months after Implantation into New Zealand White Rabbits. Heart.

[25] Qi Y., Qi H., He Y., Lin W., Li P., Qin L., Hu Y., Chen L., Liu Q., Sun H. (2018). Strategy of Metal–Polymer Composite Stent to Accelerate Biodegradation of Iron-Based Biomaterials. ACS Appl. Mater. Interfaces.

[26] Peuster M., Hesse C., Schloo T., Fink C., Beerbaum P., von Schnakenburg C. (2006). Long-Term Biocompatibility of a Corrodible Peripheral Iron Stent in the Porcine Descending Aorta. Biomaterials.

[27] Colotti G., Ilari A., Boffi A., Morea V. (2013). Metals and Metal Derivatives in Medicine. Mini-Rev. Med. Chem..

[28] Onuma Y., Ormiston J., Serruys P.W. (2011). Bioresorbable Scaffold Technologies. Circ. J..

[29] Garg S., Serruys P. (2009). Biodegradable Stents and Non-Biodegradable Stents. Minerva Cardioangiol..

[30] Morice M.-C., Serruys P.W., Sousa J.E., Fajadet J., Ban Hayashi E., Perin M., Colombo A., Schuler G., Barragan P., Guagliumi G. (2002). A Randomized Comparison of a Sirolimus-Eluting Stent with a Standard Stent for Coronary Revascularization. N. Engl. J. Med..

[31] Grube E., Silber S., Hauptmann K.E., Mueller R., Buellesfeld L., Gerckens U., Russell M.E. (2003). Six- and Twelve-Month Results from a Randomized, Double-Blind Trial on a Slow-Release Paclitaxel-Eluting Stent for De Novo Coronary Lesions. Circulation.

[32] Lüscher T.F., Steffel J., Eberli F.R., Joner M., Nakazawa G., Tanner F.C., Virmani R. (2007). Drug-Eluting Stent and Coronary Thrombosis. Circulation.

[33] Daemen J., Serruys P.W. (2007). Drug-Eluting Stent Update 2007. Circulation.

[34] Lasala J.M., Stone G.W., Dawkins K.D., Serruys P.W., Colombo A., Grube E., Koglin J., Ellis S. (2006). An Overview of the TAXUS® Express®, Paclitaxel-Eluting Stent Clinical Trial Program. J. Interv. Cardiol..

[35] Stone G.W., Ellis S.G., Cox D.A., Hermiller J., O’Shaughnessy C., Mann J.T., Turco M., Caputo R., Bergin P., Greenberg J. (2004). A Polymer-Based, Paclitaxel-Eluting Stent in Patients with Coronary Artery Disease. N. Engl. J. Med..

[36] Beijk M.A.M., Klomp M., Verouden N.J.W., van Geloven N., Koch K.T., Henriques J.P.S., Baan J., Vis M.M., Scheunhage E., Piek J.J. (2010). Genous Endothelial Progenitor Cell Capturing Stent vs. the Taxus Liberte Stent in Patients with De Novo Coronary Lesions with a High-Risk of Coronary Restenosis: A Randomized, Single-Centre, Pilot Study. Eur. Heart J..

[37] Smits P.C., Vlachojannis G.J., McFadden E.P., Royaards K.J., Wassing J., Joesoef K.S., Van Mieghem C., Van De Ent M. (2015). Final 5-Year Follow-Up of a Randomized Controlled Trial of Everolimus- and Paclitaxel-Eluting Stents for Coronary Revascularization in Daily Practice the COMPARE Trial (A Trial of Everolimus-Eluting Stents and Paclitaxel Stents for Coronary Revascularizat. JACC Cardiovasc. Interv..

[38] Fajadet J., Wijns W., Laarman G.-J., Kuck K.-H., Ormiston J., Baldus S., Hauptmann K.E., Suttorp M.J., Drzewiecki J., Pieper M. (2010). Long-Term Follow-Up of the Randomised Controlled Trial to Evaluate the Safety and Efficacy of the Zotarolimus-Eluting Driver Coronary Stent in De Novo Native Coronary Artery Lesions: Five Year Outcomes in the ENDEAVOR II Study. EuroIntervention.

[39] Kirtane A.J., Leon M.B., Ball M.W., Bajwa H.S., Sketch M.H., Coleman P.S., Stoler R.C., Papadakos S., Cutlip D.E., Mauri L. (2013). The “Final” 5-Year Follow-Up from the ENDEAVOR IV Trial Comparing a Zotarolimus-Eluting Stent with a Paclitaxel-Eluting Stent. JACC Cardiovasc. Interv..

[40] Leon M.B., Mauri L., Popma J.J., Cutlip D.E., Nikolsky E., O’Shaughnessy C., Overlie P.A., McLaurin B.T., Solomon S.L., Douglas J.S. (2010). A Randomized Comparison of the Endeavor Zotarolimus-Eluting Stent Versus the TAXUS Paclitaxel-Eluting Stent in De Novo Native Coronary Lesions: 12-Month Outcomes from the ENDEAVOR IV Trial. J. Am. Coll. Cardiol..

[41] Kandzari D.E., Mauri L., Popma J.J., Turco M.A., Gurbel P.A., Fitzgerald P.J., Leon M.B. (2011). Late-Term Clinical Outcomes with Zotarolimus- and Sirolimus-Eluting Stents: 5-Year Follow-Up of the ENDEAVOR III (a Randomized Controlled Trial of the Medtronic Endeavor Drug [ABT-578] Eluting Coronary Stent System versus the Cypher Sirolimus-Eluting Coro. JACC Cardiovasc. Interv..

[42] von Birgelen C., Basalus M.W., Tandjung K., van Houwelingen K.G., Stoel M.G., Louwerenburg J.W., Linssen G.C., Saïd S.A., Kleijne M.A., Sen H. (2012). A Randomized Controlled Trial in Second-Generation Zotarolimus-Eluting Resolute Stents Versus Everolimus-Eluting Xience V Stents in Real-World Patients: The TWENTE Trial. J. Am. Coll. Cardiol..

[43] Gada H., Kirtane A.J., Newman W., Sanz M., Hermiller J.B., Mahaffey K.W., Cutlip D.E., Sudhir K., Hou L., Koo K. (2013). 5-Year Results of a Randomized Comparison of XIENCE V Everolimus-Eluting and TAXUS Paclitaxel-Eluting Stents: Final Results From the SPIRIT III Trial (Clinical Evaluation of the XIENCE V Everolimus Eluting Coronary Stent System in the Treatment of Patient. JACC Cardiovasc. Interv..

[44] Stone G.W., Rizvi A., Sudhir K., Newman W., Applegate R.J., Cannon L.A., Maddux J.T., Cutlip D.E., Simonton C.A., Sood P. (2011). Randomized Comparison of Everolimus- and Paclitaxel-Eluting Stents: 2-Year Follow-Up from the SPIRIT (Clinical Evaluation of the XIENCE V Everolimus Eluting Coronary Stent System) IV Trial. J. Am. Coll. Cardiol..

[45] Grube E., Chevalier B., Guagliumi G., Smits P.C., Stuteville M., Dorange C., Papeleu P., Kaul U., Džavík V. (2012). The SPIRIT V Diabetic Study: A Randomized Clinical Evaluation of the XIENCE V Everolimus-Eluting Stent vs the TAXUS Liberté Paclitaxel-Eluting Stent in Diabetic Patients with De Novo Coronary Artery Lesions. Am. Heart J..

[46] Park K.W., Lee J.M., Kang S.-H., Ahn H.-S., Kang H.-J., Koo B.-K., Rhew J.Y., Hwang S.H., Lee S.Y., Kang T.S. (2014). Everolimus-Eluting Xience V/Promus Versus Zotarolimus-Eluting Resolute Stents in Patients with Diabetes Mellitus. JACC Cardiovasc. Interv..

[47] Planer D., Smits P.C., Kereiakes D.J., Kedhi E., Fahy M., Xu K., Serruys P.W., Stone G.W. (2011). Comparison of Everolimus- and Paclitaxel-Eluting Stents in Patients with Acute and Stable Coronary Syndromes: Pooled Results from the SPIRIT (A Clinical Evaluation of the XIENCE v Everolimus Eluting Coronary Stent System) and COMPARE (A Trial of Everolimu. JACC Cardiovasc. Interv..

[48] Liu S.-J., Chiang F.-J., Hsiao C.-Y., Kau Y.-C., Liu K.-S. (2010). Fabrication of Balloon-Expandable Self-Lock Drug-Eluting Polycaprolactone Stents Using Micro-Injection Molding and Spray Coating Techniques. Ann. Biomed. Eng..

[49] Chen M.C., Liu C.T., Tsai H.W., Lai W.Y., Chang Y., Sung H.W. (2009). Mechanical Properties, Drug Eluting Characteristics, and in Vivo Performance of a Genipin-Crosslinked Chitosan Polymeric Stent. Biomaterials.

[50] Bleier B.S., Paulson D.P., O’Malley B.W., Li D., Palmer J.N., Chiu A.G., Cohen N.A. (2009). Chitosan Glycerophosphate-Based Semirigid Dexamethasone Eluting Biodegradable Stent. Am. J. Rhinol. Allergy.

[51] Gogas B.D., Farooq V., Onuma Y., Serruys P.W. (2012). The ABSORB Bioresorbable Vascular Scaffold: An Evolution or Revolution in Interventional Cardiology?. Hellenic J. Cardiol..

[52] Gogas B.D. (2014). Bioresorbable Scaffolds for Percutaneous Coronary Interventions. Glob. Cardiol. Sci. Pract..

[53] Mattesini A., Bartolini S., Sorini Dini C., Valente S., Parodi G., Stolcova M., Meucci F., Di Mario C. (2017). The DESolve Novolimus Bioresorbable Scaffold: From Bench to Bedside. J. Thorac. Dis..

[54] Verheye S., Ormiston J.A., Stewart J., Webster M., Sanidas E., Costa R., Costa J.R.J., Chamie D., Abizaid A.S., Pinto I. (2014). A Next-Generation Bioresorbable Coronary Scaffold System: From Bench to First Clinical Evaluation: 6- and 12-Month Clinical and Multimodality Imaging Results. JACC Cardiovasc. Interv..

[55] Iqbal J., Onuma Y., Ormiston J., Abizaid A., Waksman R., Serruys P. (2014). Bioresorbable Scaffolds: Rationale, Current Status, Challenges, and Future. Eur. Heart J..

[56] Iqbal J., Verheye S., Abizaid A., Ormiston J., de Vries T., Morrison L., Toyloy S., Fitzgerald P., Windecker S., Serruys P.W. (2016). DESyne Novolimus-Eluting Coronary Stent Is Superior to Endeavor Zotarolimus-Eluting Coronary Stent at Five-Year Follow-Up: Final Results of the Multicentre EXCELLA II Randomised Controlled Trial. EuroIntervention.

[57] Boeder N.F., Dorr O., Bauer T., Mattesini A., Elsasser A., Liebetrau C., Achenbach S., Hamm C.W., Nef H.M. (2017). Impact of Strut Thickness on Acute Mechanical Performance: A Comparison Study Using Optical Coherence Tomography between DESolve 150 and DESolve 100. Int. J. Cardiol..

[58] Ramcharitar S., Serruys P.W. (2008). Fully Biodegradable Coronary Stents: Progress to Date. Am. J. Cardiovasc. Drugs.

[59] Abizaid A., Carrie D., Frey N., Lutz M., Weber-Albers J., Dudek D., Chevalier B., Weng S.-C., Costa R.A., Anderson J. (2017). 6-Month Clinical and Angiographic Outcomes of a Novel Radiopaque Sirolimus-Eluting Bioresorbable Vascular Scaffold: The FANTOM II Study. JACC Cardiovasc. Interv..

[60] Zhang Y., Bourantas C.V., Farooq V., Muramatsu T., Diletti R., Onuma Y., Garcia-Garcia H.M., Serruys P.W. (2013). Bioresorbable Scaffolds in the Treatment of Coronary Artery Disease. Med. Devices.

[61] Rao A.S., Makaroun M.S., Marone L.K., Cho J.S., Rhee R., Chaer R.A. (2011). Long-Term Outcomes of Internal Carotid Artery Dissection. J. Vasc. Surg..

[62] Kumar A.S., Hariram V. (2014). Indigenous Stents: Examining the Clinical Data on New Technologies. Interv. Cardiol..

[63] Cheng Y., Gasior P., Shibuya M., Ramzipoor K., Lee C., Estrada E.A., Dokko D., McGregor J.C., Conditt G.B., Kaluza G.L. (2016). Comparative Characterization of Biomechanical Behavior and Healing Profile of a Novel Ultra-High-Molecular-Weight Amorphous Poly-l-Lactic Acid Sirolimus-Eluting Bioresorbable Coronary Scaffold. Circ. Cardiovasc. Interv..

[64] Indolfi C., De Rosa S., Colombo A. (2016). Bioresorbable Vascular Scaffolds—Basic Concepts and Clinical Outcome. Nat. Rev. Cardiol..

[65] Wu Y., Shen L., Wang Q., Ge L., Xie J., Hu X., Sun A., Qian J., Ge J. (2012). Comparison of Acute Recoil between Bioabsorbable Poly-L-Lactic Acid XINSORB Stent and Metallic Stent in Porcine Model. J. Biomed. Biotechnol..

[66] Shen L., Wu Y., Ge L., Zhang Y., Wang Q., Qian J., Qiu Z., Ge J. (2017). A Head to Head Comparison of XINSORB Bioresorbable Sirolimus-Eluting Scaffold versus Metallic Sirolimus-Eluting Stent: 180 Days Follow-Up in a Porcine Model. Int. J. Cardiovasc. Imaging.

[67] Wang Y., Zhang X. (2014). Vascular Restoration Therapy and Bioresorbable Vascular Scaffold. Regen. Biomater..

[68] Muramatsu T., Onuma Y., Zhang Y.-J., Bourantas C.V., Kharlamov A., Diletti R., Farooq V., Gogas B.D., Garg S., Garcia-Garcia H.M. (2013). Progress in Treatment by Percutaneous Coronary Intervention: The Stent of the Future. Rev. Esp. Cardiol..

[69] Jabara R., Pendyala L., Geva S., Chen J., Chronos N., Robinson K. (2009). Novel Fully Bioabsorbable Salicylate-Based Sirolimus-Eluting Stent. EuroIntervention.

[70] Tenekecioglu E., Farooq V., Bourantas C.V., Silva R.C., Onuma Y., Yilmaz M., Serruys P.W. (2016). Bioresorbable Scaffolds: A New Paradigm in Percutaneous Coronary Intervention. BMC Cardiovasc. Disord..

[71] Costa R.A., Liew H.-B., Abizaid A., de Ribamar Costa J., Chamié D., Abizaid A., Castro J.P., Serruys P.W., Santoso T. (2015). TCT-546 6-Month Angiographic Results of the Novel MIRAGE Microfiber Sirolimus-Eluting Bioresorbable Vascular Scaffold—A Quantitative Coronary Angiography Analysis from the Prospective, Randomized MIRAGE Clinical Trial. J. Am. Coll. Cardiol..

[72] Hideo T., Keiji I., Eisho K., Kunihiko K., Akiyoshi K., Shigeo M., Hidenori K., Takafumi T., Seiichiro M., Hiromu U. (2000). Initial and 6-Month Results of Biodegradable Poly-l-Lactic Acid Coronary Stents in Humans. Circulation.

[73] Nishio S., Kosuga K., Igaki K., Okada M., Kyo E., Tsuji T., Takeuchi E., Inuzuka Y., Takeda S., Hata T. (2012). Long-Term (>10 Years) Clinical Outcomes of First-in-Human Biodegradable Poly-l-Lactic Acid Coronary Stents: Igaki-Tamai Stents. Circulation.

[74] Yahagi K., Yang Y., Torii S., Mensah J., White R.M., Mathieu M., Pacheco E., Nakano M., Barakat A., Sharkawi T. (2017). Comparison of a Drug-Free Early Programmed Dismantling PDLLA Bioresorbable Scaffold and a Metallic Stent in a Porcine Coronary Artery Model at 3-Year Follow-Up. J. Am. Heart Assoc..

[75] Durand E., Sharkawi T., Leclerc G., Raveleau M., van der Leest M., Vert M., Lafont A. (2014). Head-to-Head Comparison of a Drug-Free Early Programmed Dismantling Polylactic Acid Bioresorbable Scaffold and a Metallic Stent in the Porcine Coronary Artery: Six-Month Angiography and Optical Coherence Tomographic Follow-Up Study. Circ. Cardiovasc. Interv..

[76] Ielasi A., Tespili M. (2014). Current and Future Perspectives on Drug-Eluting Bioresorbable Coronary Scaffolds. Future Cardiol..

[77] Kenny D., Hijazi Z.M. (2015). Bioresorbable Stents for Pediatric Practice: Where Are We Now?. Interv. Cardiol..

[78] Hoffmann R., Mintz G.S., Dussaillant G.R., Popma J.J., Pichard A.D., Satler L.F., Kent K.M., Griffin J., Leon M.B. (1996). Patterns and Mechanisms of In-Stent Restenosis. Circulation.

[79] Haude M., Ince H., Abizaid A., Toelg R., Lemos P.A., von Birgelen C., Christiansen E.H., Wijns W., Neumann F.-J., Kaiser C. (2016). Safety and Performance of the Second-Generation Drug-Eluting Absorbable Metal Scaffold in Patients with de-Novo Coronary Artery Lesions (BIOSOLVE-II): 6 Month Results of a Prospective, Multicentre, Non-Randomised, First-in-Man Trial. Lancet.

[80] Haude M., Erbel R., Erne P., Verheye S., Degen H., Vermeersch P., Weissman N., Prati F., Bruining N., Waksman R. (2016). Safety and Performance of the DRug-Eluting Absorbable Metal Scaffold (DREAMS) in Patients with De Novo Coronary Lesions: 3-Year Results of the Prospective, Multicentre, First-in-Man BIOSOLVE-I Trial. EuroIntervention.

[81] Haddad R.N., Adel Hassan A., Al Soufi M., Kasem M. (2023). A Word of Caution: Early Failure of Magmaris® Bioresorbable Stent after Pulmonary Artery Stenting. Catheter. Cardiovasc. Interv..

[82] He Y., Wang J., Yan W., Huang N. (2014). Gallic Acid and Gallic Acid-Loaded Coating Involved in Selective Regulation of Platelet, Endothelial and Smooth Muscle Cell Fate. RSC Adv..

[83] Toyota T., Morimoto T., Shiomi H., Yoshikawa Y., Yaku H., Yamashita Y., Kimura T. (2017). Very Late Scaffold Thrombosis of Bioresorbable Vascular Scaffold: Systematic Review and a Meta-Analysis. JACC Cardiovasc. Interv..

[84] Verschuur E.M.L., Steyerberg E.W., Kuipers E.J., Siersema P.D. (2007). Effect of Stent Size on Complications and Recurrent Dysphagia in Patients with Esophageal or Gastric Cardia Cancer. Gastrointest. Endosc..

[85] Ntiamoah P., Gildea T.R., Baiera A. (2023). Determination of Patient-Specific Airway Stent Fit Using Novel 3D Reconstruction Measurement Techniques: A 4-Year Follow-Up of a Patient. Ther. Adv. Respir. Dis..

[86] Alexy R.D., Levi D.S. (2013). Materials and Manufacturing Technologies Available for Production of a Pediatric Bioabsorbable Stent. Biomed. Res. Int..

[87] Martinez A.W., Chaikof E.L. (2011). Microfabrication and Nanotechnology in Stent Design. WIREs Nanomed. Nanobiotechnol..

[88] Ballyns J.J., Gleghorn J.P., Niebrzydowski V., Rawlinson J.J., Potter H.G., Maher S.A., Wright T.M., Bonassar L.J. (2008). Image-Guided Tissue Engineering of Anatomically Shaped Implants via MRI and Micro-CT Using Injection Molding. Tissue Eng. Part. A.

[89] Bartolo P., Kruth J.P., Silva J., Levy G., Malshe A., Rajurkar K., Mitsuishi M., Ciurana J., Leu M. (2012). Biomedical Production of Implants by Additive Electro-Chemical and Physical Processes. CIRP Ann. Manuf. Technol..

[90] Hung K.C., Tseng C.S., Hsu S.H. (2014). Synthesis and 3D Printing of Biodegradable Polyurethane Elastomer by a Water-Based Process for Cartilage Tissue Engineering Applications. Adv. Healthc. Mater..

[91] De Leon A.C., Chen Q., Palaganas N.B., Palaganas J.O., Manapat J., Advincula R.C. (2016). High Performance Polymer Nanocomposites for Additive Manufacturing Applications. React. Funct. Polym..

[92] Wong K.V., Hernandez A. (2012). A Review of Additive Manufacturing. ISRN Mech. Eng..

[93] Mohamed O.A., Masood S.H., Bhowmik J.L. (2015). Optimization of Fused Deposition Modeling Process Parameters: A Review of Current Research and Future Prospects. Adv. Manuf..

[94] Korpela J., Kokkari A., Korhonen H., Malin M., Närhi T., Seppälä J. (2013). Biodegradable and Bioactive Porous Scaffold Structures Prepared Using Fused Deposition Modeling. J. Biomed. Mater. Res. B Appl. Biomater..

[95] Konta A., García M., Serrano D. (2017). Personalised 3D Printed Medicines: Which Techniques and Polymers Are More Successful?. Bioengineering.

[96] Jakus A.E., Taylor S.L., Geisendorfer N.R., Dunand D.C., Shah R.N. (2015). Metallic Architectures from 3D-Printed Powder-Based Liquid Inks. Adv. Funct. Mater..

[97] Olga I., Christopher W., Thomas C. (2013). Additive Manufacturing (AM) and Nanotechnology: Promises and Challenges. Rapid Prototyp. J..

[98] Misra S.K., Ostadhossein F., Babu R., Kus J., Tankasala D., Sutrisno A., Walsh K.A., Bromfield C.R., Pan D. (2017). 3D-Printed Multidrug-Eluting Stent from Graphene-Nanoplatelet-Doped Biodegradable Polymer Composite. Adv. Healthc. Mater..

[99] Cui J., Li J., Mathison M., Tondato F., Mulkey S.P., Micko C., Chronos N.A.F., Robinson K.A. (2005). A Clinically Relevant Large-Animal Model for Evaluation of Tissue-Engineered Cardiac Surgical Patch Materials. Cardiovasc. Revascularization Med..

[100] Jia H., Gu S.Y., Chang K. (2018). 3D Printed Self-Expandable Vascular Stents from Biodegradable Shape Memory Polymer. Adv. Polym. Technol..

[101] Alam J., Khan A., Alam M., Mohan R. (2015). Electroactive Shape Memory Property of a Cu-Decorated CNT Dispersed PLA/ESO Nanocomposite. Materials.

[102] Wu Z., Zhao J., Wu W., Wang P., Wang B., Li G., Zhang S. (2018). Radial Compressive Property and the Proof-of-Concept Study for Realizing Self-Expansion of 3D Printing Polylactic Acid Vascular Stents with Negative Poisson’s Ratio Structure. Materials.

[103] Wang W.-Q., Liang D.-K., Yang D.-Z., Qi M. (2006). Analysis of the Transient Expansion Behavior and Design Optimization of Coronary fStents by Finite Element Method. J. Biomech..

[104] Wang C., Zhang L., Fang Y., Sun W. (2020). Design, Characterization, and 3D Printing of Cardiovascular Stents with Zero Poisson’s Ratio in Longitudinal Deformation. Engineering.

[105] Guerra A., Roca A., de Ciurana J. (2017). A Novel 3D Additive Manufacturing Machine to Biodegradable Stents. Procedia Manuf..

[106] Guerra A.J., Cano P., Rabionet M., Puig T., Ciurana J. (2018). 3D-Printed PCL/PLA Composite Stents: Towards a New Solution to Cardiovascular Problems. Materials.

[107] Qiu T., Jiang W., Yan P., Jiao L., Wang X. (2020). Development of 3D-Printed Sulfated Chitosan Modified Bioresorbable Stents for Coronary Artery Disease. Front. Bioeng. Biotechnol..

[108] Lutter C., Nothhaft M., Rzany A., Garlichs C.D., Cicha I. (2015). Effect of Specific Surface Microstructures on Substrate Endothelialisation and Thrombogenicity: Importance for Stent Design. Clin. Hemorheol. Microcirc..

[109] Vurugonda U., Rednam P., Sinha M. (2018). Development of Biodegradable Scaffold Using Polylactic Acid and Polycaprolactone for Cardiovascular Application. Int. J. Polym. Mater. Polym. Biomater..

[110] Zamiri P., Kuang Y., Sharma U., Ng T.F., Busold R.H., Rago A.P., Core L.A., Palasis M. (2010). The Biocompatibility of Rapidly Degrading Polymeric Stents in Porcine Carotid Arteries. Biomaterials.

[111] Martins J.A., Lach A.A., Morris H.L., Carr A.J., Mouthuy P.-A. (2020). Polydioxanone Implants: A Systematic Review on Safety and Performance in Patients. J. Biomater. Appl..

[112] Chen E., Xiong Z., Cai X., Liu S., Qin X., Sun J., Jin X., Sun K. (2023). Bioresorbable PPDO Sliding-Lock Stents with Optimized FDM Parameters for Congenital Heart Disease Treatment. J. Mech. Behav. Biomed. Mater..

[113] Okereke M.I., Khalaj R., Tabriz A.G., Nandi U., Scoutaris N., Douroumis D. (2023). Development of 3D Printable Bioresorbable Drug Eluting Coronary Stents: An Experimental and Computational Investigation. J. Drug Deliv. Sci. Technol..

[114] Singh J., Pandey P.M., Kaur T., Singh N. (2021). A Comparative Analysis of Solvent Cast 3D Printed Carbonyl Iron Powder Reinforced Polycaprolactone Polymeric Stents for Intravascular Applications. J. Biomed. Mater. Res. B Appl. Biomater..

[115] Singh J., Kaur T., Singh N., Pandey P.M. (2020). Biological and Mechanical Characterization of Biodegradable Carbonyl Iron Powder/Polycaprolactone Composite Material Fabricated Using Three-Dimensional Printing for Cardiovascular Stent Application. Proc. Inst. Mech. Eng. Part H J. Eng. Med..

[116] Singh J., Singh G., Pandey P.M. (2021). Multi-Objective Optimization of Solvent Cast 3D Printing Process Parameters for Fabrication of Biodegradable Composite Stents. Int. J. Adv. Manuf. Technol..

[117] van Lith R., Baker E., Ware H., Yang J., Farsheed A.C., Sun C., Ameer G. (2016). 3D-Printing Strong High-Resolution Antioxidant Bioresorbable Vascular Stents. Adv. Mater. Technol..

[118] Tumbleston J.R., Shirvanyants D., Ermoshkin N., Janusziewicz R., Johnson A.R., Kelly D., Chen K., Pinschmidt R., Rolland J.P., Ermoshkin A. (2015). Continuous Liquid Interface Production of 3D Objects. Science.

[119] Park S.A., Lee S.J., Lim K.S., Bae I.H., Lee J.H., Kim W.D., Jeong M.H., Park J.K. (2015). In Vivo Evaluation and Characterization of a Bio-Absorbable Drug-Coated Stent Fabricated Using a 3D-Printing System. Mater. Lett..

[120] Flege C., Vogt F., Höges S., Jauer L., Borinski M., Schulte V.A., Hoffmann R., Poprawe R., Meiners W., Jobmann M. (2013). Development and Characterization of a Coronary Polylactic Acid Stent Prototype Generated by Selective Laser Melting. J. Mater. Sci. Mater. Med..

[121] Ware H.O.T., Farsheed A.C., van Lith R., Baker E., Ameer G., Sun C. (2017). Process Development for High-Resolution 3D-Printing of Bioresorbable Vascular Stents. Advanced Fabrication Technologies for Micro/Nano Optics and Photonics X.

[122] de Oliveira M.F., da Silva L.C.E., de Oliveira M.G. (2021). 3D Printed Bioresorbable Nitric Oxide-Releasing Vascular Stents. Bioprinting.

[123] Napoli C., Paolisso G., Casamassimi A., Al-Omran M., Barbieri M., Sommese L., Infante T., Ignarro L.J. (2013). Effects of Nitric Oxide on Cell Proliferation: Novel Insights. J. Am. Coll. Cardiol..

[124] Rajendran P., Rengarajan T., Thangavel J., Nishigaki Y., Sakthisekaran D., Sethi G., Nishigaki I. (2013). The Vascular Endothelium and Human Diseases. Int. J. Biol. Sci..

[125] Xu J., Zou M.-H. (2009). Molecular Insights and Therapeutic Targets for Diabetic Endothelial Dysfunction. Circulation.

[126] Böger R.H. (2007). The Pharmacodynamics of L-Arginine. J. Nutr..

[127] Lei J., Vodovotz Y., Tzeng E., Billiar T.R. (2013). Nitric Oxide, a Protective Molecule in the Cardiovascular System. Nitric Oxide.

[128] Cha W., Meyerhoff M.E. (2007). Catalytic Generation of Nitric Oxide from S-Nitrosothiols Using Immobilized Organoselenium Species. Biomaterials.

[129] Yang Z., Yang Y., Xiong K., Li X., Qi P., Tu Q., Jing F., Weng Y., Wang J., Huang N. (2015). Nitric Oxide Producing Coating Mimicking Endothelium Function for Multifunctional Vascular Stents. Biomaterials.

[130] Deux J.F., Meddahi-Pellé A., Bree F., Bataille I., Michel J.B., Letourneur D. (2009). Comparative Studies on the Mechanisms of Action of Four Polysaccharides on Arterial Restenosis. J. Biomater. Sci. Polym. Ed..

[131] Deux J.-F., Meddahi-Pellé A., Le Blanche A.F., Feldman L.J., Colliec-Jouault S., Brée F., Boudghène F., Michel J.-B., Letourneur D. (2002). Low Molecular Weight Fucoidan Prevents Neointimal Hyperplasia in Rabbit Iliac Artery In-Stent Restenosis Model. Arterioscler. Thromb. Vasc. Biol..

[132] Kim J.M., Bae I.-H., Lim K.S., Park J.-K., Park D.S., Lee S.-Y., Jang E.-J., Ji M.S., Sim D.S., Hong Y.J. (2015). A Method for Coating Fucoidan onto Bare Metal Stent and In Vivo Evaluation. Prog. Org. Coat..

[133] Roux N., Brakenhielm E., Freguin-Bouillant C., Lallemand F., Henry J.-P., Boyer O., Thuillez C., Plissonnier D. (2012). Progenitor Cell Mobilizing Treatments Prevent Experimental Transplant Arteriosclerosis. J. Surg. Res..

[134] Keuren J.F.W., Wielders S.J.H., Willems G.M., Morra M., Cahalan L., Cahalan P., Lindhout T. (2003). Thrombogenicity of Polysaccharide-Coated Surfaces. Biomaterials.

[135] Thalla P.K., Fadlallah H., Liberelle B., Lequoy P., De Crescenzo G., Merhi Y., Lerouge S. (2014). Chondroitin Sulfate Coatings Display Low Platelet but High Endothelial Cell Adhesive Properties Favorable for Vascular Implants. Biomacromolecules.

[136] Verheye S., Markou C.P., Salame M.Y., Wan B., King S.B., Robinson K.A., Chronos N.A.F., Hanson S.R. (2000). Reduced Thrombus Formation by Hyaluronic Acid Coating of Endovascular Devices. Arterioscler. Thromb. Vasc. Biol..

[137] Li G., Yang P., Qin W., Maitz M.F., Zhou S., Huang N. (2011). The Effect of Coimmobilizing Heparin and Fibronectin on Titanium on Hemocompatibility and Endothelialization. Biomaterials.

[138] Boyle J.P., Smart R.H., Shirey J.K. (1964). Heparin in the Treatment of Chronic Obstructive Bronchopulmonary Disease. Am. J. Cardiol..

[139] Lee S.J., Jo H.H., Lim K.S., Lim D., Lee S., Lee J.H., Kim W.D., Jeong M.H., Lim J.Y., Kwon I.K. (2019). Heparin Coating on 3D Printed Poly (l-Lactic Acid) Biodegradable Cardiovascular Stent via Mild Surface Modification Approach for Coronary Artery Implantation. Chem. Eng. J..

[140] Liu Z., Zheng Z., Chen K., Li Y., Wang X., Li G. (2019). A Heparin-Functionalized Woven Stent Graft for Endovascular Exclusion. Colloids Surf. B Biointerfaces.

[141] Luo C., Zheng Y., Diao Z., Qiu J., Wang G. (2011). Review: Research Progress and Future Prospects for Promoting Endothelialization on Endovascular Stents and Preventing Restenosis. J. Med. Biol. Eng..

[142] Wawrzyńska M., Duda M., Wysokińska E., Strządała L., Biały D., Ulatowska-Jarża A., Kałas W., Kraszewski S., Pasławski R., Biernat P. (2019). Functionalized CD133 Antibody Coated Stent Surface Simultaneously Promotes EPCs Adhesion and Inhibits Smooth Muscle Cell Proliferation–A Novel Approach to Prevent in-Stent Restenosis. Colloids Surf. B Biointerfaces.

[143] Wu X., Yin T., Tian J., Tang C., Huang J., Zhao Y., Zhang X., Deng X., Fan Y., Yu D. (2015). Distinctive Effects of CD34- and CD133-Specific Antibody-Coated Stents on Re-Endothelialization and in-Stent Restenosis at the Early Phase of Vascular Injury. Regen. Biomater..

[144] Xiao L., Wang G., Jiang T., Tang C., Wu X., Sun T. (2011). Effects of Shear Stress on the Number and Function of Endothelial Progenitor Cells Adhered to Specific Matrices. J. Appl. Biomater. Biomech..

[145] Chausse V., Mas-Moruno C., Martin-Gómez H., Pino M., Díaz-Ricart M., Escolar G., Ginebra M.-P., Pegueroles M. (2023). Functionalization of 3D Printed Polymeric Bioresorbable Stents with a Dual Cell-Adhesive Peptidic Platform Combining RGDS and YIGSR Sequences. Biomater. Sci..

[146] Valadi H., Ekström K., Bossios A., Sjöstrand M., Lee J.J., Lötvall J.O. (2007). Exosome-Mediated Transfer of MRNAs and MicroRNAs Is a Novel Mechanism of Genetic Exchange between Cells. Nat. Cell Biol..

[147] Hou Y.-C., Li J.-A., Zhu S.-J., Cao C., Tang J.-N., Zhang J.-Y., Guan S.-K. (2020). Tailoring of Cardiovascular Stent Material Surface by Immobilizing Exosomes for Better Pro-Endothelialization Function. Colloids Surf. B Biointerfaces.

[148] Luo L., Tang J., Nishi K., Yan C., Dinh P.-U., Cores J., Kudo T., Zhang J., Li T.-S., Cheng K. (2017). Fabrication of Synthetic Mesenchymal Stem Cells for the Treatment of Acute Myocardial Infarction in Mice. Circ. Res..

[149] Gong M., Yu B., Wang J., Wang Y., Liu M., Paul C., Millard R.W., Xiao D.S., Ashraf M., Xu M. (2017). Mesenchymal Stem Cells Release Exosomes That Transfer MiRNAs to Endothelial Cells and Promote Angiogenesis. Oncotarget.

